# Root Suberin Plays Important Roles in Reducing Water Loss and Sodium Uptake in *Arabidopsis thaliana*

**DOI:** 10.3390/metabo11110735

**Published:** 2021-10-27

**Authors:** Nayana D. G. de Silva, Jhadeswar Murmu, Denise Chabot, Keith Hubbard, Peter Ryser, Isabel Molina, Owen Rowland

**Affiliations:** 1 Department of Biology and Institute of Biochemistry, Carleton University, Ottawa, ON K1S 5B6, Canada; nayanadesilva@cmail.carleton.ca (N.D.G.d.S.); jhadeswarmurmu@cunet.carleton.ca (J.M.); 2 Agriculture and Agri-Food Canada, Ottawa Research and Development Centre, Ottawa, ON K1A 0C6, Canada; denise.chabot@canada.ca (D.C.); keith.hubbard@canada.ca (K.H.); 3 Department of Biology, Laurentian University, Sudbury, ON P3E 2C6, Canada; pryser@laurentian.ca; 4 Department of Biology, Algoma University, Sault Ste. Marie, ON P6A 2G4, Canada

**Keywords:** suberin, waxes, chronic drought, salt stress, root periderm, K/Na ratio

## Abstract

Suberin is a cell-wall-associated hetero-polymer deposited in specific plant tissues. The precise role of its composition and lamellae structure in protecting plants against abiotic stresses is unclear. In *Arabidopsis thaliana*, we tested the biochemical and physiological responses to water deficiency and NaCl treatment in mutants that are differentially affected in suberin composition and lamellae structure. Chronic drought stress increased suberin and suberin-associated waxes in wild-type plants. Suberin-deficient mutants were not more susceptible than the wild-type to the chronic drought stress imposed in this study. Nonetheless, the *cyp86a1-1 cyp86b1-1* mutant, which had a severely altered suberin composition and lamellae structure, exhibited increased water loss through the root periderm. *Cyp86a1-1 cyp86b1-1* also recorded lower relative water content in leaves. The *abcg2-1 abcg6-1 abcg20-1* mutant, which has altered suberin composition and lamellae, was very sensitive to NaCl treatment. Furthermore, *cyp86a1-1 cyp86b1-1* recorded a significant drop in the leaf K/Na ratio, indicating salt sensitivity. The *far1-2 far4-1 far5-1* mutant, which did not show structural defects in the suberin lamellae, had similar responses to drought and NaCl treatments as the wild-type. Our results provide evidence that the suberin amount and lamellae structure are key features in the barrier function of suberin in reducing water loss and reducing sodium uptake through roots for better performance under drought and salt stresses.

## 1. Introduction 

Resistance to drought and high salinity have been the main foci in crop improvement efforts due to reductions in crop productivity caused by climate change [1]. Drought can be chronic in climatic regions with overall low water availability or acute in situations where weather conditions change rapidly [2]. Chronic water deficiency in soil is often linked to high salinity, which is also symptomatic of general soil degradation. Salt stress, usually dominated by sodium chloride (NaCl) toxicity [3], can impact plant growth and development, ultimately affecting crop yields. Thus, a better understanding of chronic drought stress and high NaCl soil concentrations in relation to plant health is of great importance for sustainable agriculture.

Being sessile, plants have evolved multiple acclimation and adaptation mechanisms to respond to environmental stresses. Analysis of these protective mechanisms will contribute to our knowledge of plant tolerance to stress conditions. Plants have various hydrophobic barriers that provide protection against water loss. Suberin is one such cell-wall-associated barrier located in certain tissues, such as the root endodermis and periderm [4]. Suberin typically presents a lamellar structure when observed by transmission electron microscopy (TEM). Two views have been presented for the structure of suberin. Bernards [5] proposed that suberin consists of a distinct ester-bonded poly-aliphatic domain (forming the lamellae) that is attached to a cross-linked poly-aromatic domain. Alternatively, suberin is referred to as the poly (acylglycerol) macromolecule, corresponding to the electron-translucent lamellae, which is cross-linked to lignin-like polyaromatics, forming the dark lamellae [6]. In *Arabidopsis thaliana*, the poly-aliphatic domain contains long-chain and very-long-chain fatty acids, ω-hydroxy fatty acids, α, ω-dicarboxylic fatty acids, and primary fatty alcohols, as well as glycerol and ferulic acid [6]. The most abundant monomer groups are α, ω-dicarboxylic fatty acids (DCAs) and ω-hydroxy fatty acids (ω-OH FAs), which represent 24% and 43% of the aliphatic monomer content, respectively, in roots of 5-week-old Arabidopsis plants [7]. The suberin polymer is thought to be embedded in waxes that are non-polymeric [8,9]. In Arabidopsis root waxes, the major components are alkyl hydroxycinnamates (AHCs) [10], particularly alkyl coumarates and alkyl caffeates [8,9].

In Arabidopsis, root suberin deposition changes through plant development [11,12,13]. During primary growth, suberin is deposited primarily in the endodermis. Subsequently, tissues that undergo secondary growth form a periderm that replaces the epidermis and becomes the outermost tissue that comes in contact with the external environment. A mature periderm is observed in portions of the primary root of Arabidopsis as early as 14 days after sowing [12].

Suberin deposition can also be modulated in response to different environmental stresses. North and Nobel [14] reported that a decrease in root hydraulic conductivity correlates with increased suberization in tropical epiphytic cacti roots under drought stress. Similarly, in response to osmotic stress, genes in the suberin biosynthesis pathway are upregulated to increase suberin in the endodermis and decrease hydraulic conductivity in barley roots [15]. Studies in rice (*Oryza sativa*) revealed the induction of suberin deposition under salt stress [16] and oxygen deficiency [17]. Additionally, in rice, high ammonium levels increase root suberin content and decrease solute permeability [18]. An increase in root suberization in rice was correlated with a decrease in shoot Na content under salt stress [19]. Suberin biosynthesis is increased by salt treatment in *Avicennia officinalis* roots [20], by waterlogged conditions in rice and maize [21,22], and by soil nutrient deficiency in castor bean and Arabidopsis [23,24]. Franke et al. [25] reported that root suberin in 5-week-old Arabidopsis plants is increased by 100 mM NaCl salt stress compared to control plants. A knock-out mutation in Arabidopsis *CYP86A1*, which encodes a fatty acid ω-hydroxylase involved in suberin monomer biosynthesis, results in higher root hydraulic conductivity than the wild type [26,27]. The Arabidopsis mutant *esb1* has ectopic suberin deposition in roots. Compared to the wild type, *esb1* has higher water-use efficiency (WUE) and a lower transpiration rate associated with enhanced suberin deposition in roots [28]. Conversely, suberin-deficient mutants show susceptibility to salt stress [24,29,30]. In a recent study, suberin deposition was characterized through development and found to be upregulated under water-deficit conditions in grapevine roots [31].

Although the above-described studies imply a relationship between suberization and physiological parameters in plants that relate to water and solute uptake, there is still a gap in our understanding of the role of suberin composition and ultrastructure in tolerance to drought or salinity. We hypothesized that Arabidopsis mutants altered in suberin composition and ultrastructure would have altered water relations and altered solute uptake in roots. Mutants selected in this study were affected in suberin monomer transport (*abcg2-1 abcg6-1 abcg20-1*), suberin biosynthesis (*far1-2 far4-1 far5-1* and *cyp86a1-1 cyp86b1-1*), or regulation of suberin synthesis (*myb92-1 myb93-1*). Compared to the wild type, roots of the triple *abcg2-1 abcg6-1 abcg20-1* mutant roots have a distorted suberin lamellae structure and major reductions in alkyl hydroxycinnamates of suberin-associated waxes [32]. However, the total suberin aliphatic monomer content of *abcg2-1 abcg6-1 abcg20-1* is 70% higher than the wild type in roots of 10-day-old seedlings and three times higher than the wild type in roots from 7-week-old plants [32]. The mature root periderm of *abcg2-1 abcg6-1 abcg20-1* is reduced in 20:0 and 22:0 fatty acids and 22:0 fatty alcohol [32]. Further, it has a 33% decrease in 18:1 ω-OH-FA compared to the wild type [32]. Additionally, the root system of this *ABCG* triple mutant was reported to be more permeable to water and salts [32]. Mutations in *FAR1*, *FAR4*, and *FAR5* cause reductions in 22:0-OH, 20:0-OH, and 18:0-OH, respectively, and triple *far1 far4 far5* mutants have a 70% overall reduction in primary alcohol content in the suberin polymer (all chain lengths affected) and a greater than 90% reduction in alkyl hydroxycinnamates in the suberin-associated waxes [10]. Individual knock-out mutations in *CYP86A1* and *CYP86B1* result in strong reductions in C16 and C18 [11] and C22 and C24 [33] chain-length ω-OH FAs and DCAs, respectively. However, the double mutant *cyp86a1-1 cyp86b1-1* has not been previously reported and our data showed that it is reduced in all chain lengths of DCAs and ω-OH FA monomers and thus is expected to have a major perturbation of suberin structure and function. Additionally, the double mutant *myb92-1 myb93-1* was included in this study because our preliminary studies showed that compared to the single mutants (*myb92-1* and *myb93-1*), the double mutant has reduced amounts of all suberin monomers. Further, apple MYB93 is thought to play a major role in the regulation of suberin deposition in russeted apple skins [34], and transient expression of Arabidopsis MYB92 enhances suberin deposition in the leaves of *Nicotiana benthamiana* [35].

In previous studies, the implementation of acute drought stress was the most common method in investigating drought stress responses in plants. However, such studies have limited relevance in the development of drought-resistant crops [36]. Therefore, a chronic drought assay was implemented in this study. By investigating the chemical and phenotypic responses to chronic drought and NaCl stresses in our Arabidopsis mutant collection, we report that suberin composition and lamellae structure are important to reduce water loss through the root periderm and to restrict uncontrolled Na movement to the shoot through the roots. These features help plants perform better under drought and high-salinity conditions.

## 2. Results

### 2.1. Chronic Drought Stress Induces Suberin and Suberin-Associated Waxes in Wild-Type Plants

**Figure 1:**
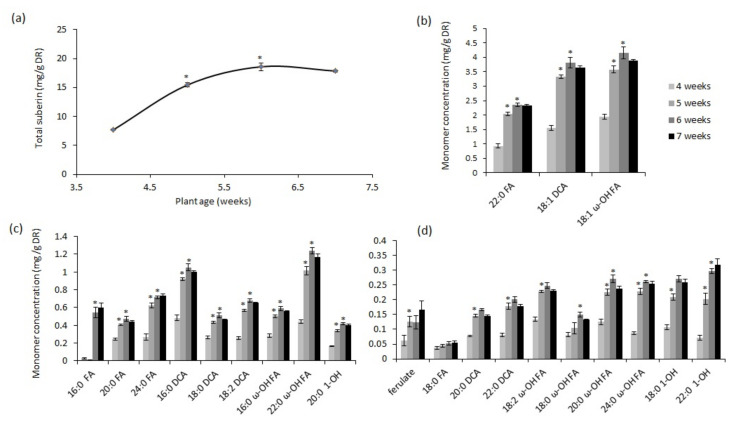
Time-course analysis of Arabidopsis root suberin through weeks 4 to 7 of development. (**a**) Total poly-aliphatic suberin content, (**b**) most-abundant suberin monomers, (**c**) moderately abundant suberin monomers, and (**d**) least-abundant suberin monomers in roots of wild-type (Col-0) plants at different ages (4–7 weeks) grown under a constant, non-drought condition (20% water level). Data points are mean values in milligrams per gram of delipidated dry residue (DR) from five replicate samples ± SE. Each replicate represented a pool of roots from 4–5 plants. FA, fatty acid; DCA, dicarboxylic acid; ω-OH FA, omega hydroxy fatty acid; 1-OH, primary alcohol. Asterisks indicate significance at *p* < 0.05 by Student’s *t*-test compared to the suberin content of the previous week.

A time-course of poly-aliphatic suberin production in roots was analyzed using 4- to 7-week-old wild-type Arabidopsis plants to better understand how developmental suberin biosynthesis, which is that produced normally under control growth conditions, is regulated under non-drought conditions (20% *v*/*v* water content). Earlier time points were not included in our experiment because these have been previously reported [9,11] and because 4-week-old plants and older are the relevant plant ages for the chronic drought and salt stress experiments described below. Under non-drought conditions, the total root suberin content in the wild type increased between 4 and 6 weeks of age, after which suberin production reached a plateau (Figure 1a). The highest rate of suberin production was observed between weeks 4 and 5, when it nearly doubled in one week (Figure 1a). This was due to increases in its most abundant monomers, α, ω-octadecene dioic acid (18:1 DCA), ω-hydroxy octadecenoic fatty acid (18:1 ω-OH FA), and docosanoic fatty acid (22:0 FA) (Figure 1b), as well as increases in the majority of the less-abundant monomers (Figure 1b,d). Whereas ferulate reached its maximum amount at 5 weeks, the aliphatic monomers reached maximum amounts at 6 weeks after germination, coinciding with the highest total suberin accumulation (Figure 1d).

**Figure 2:**
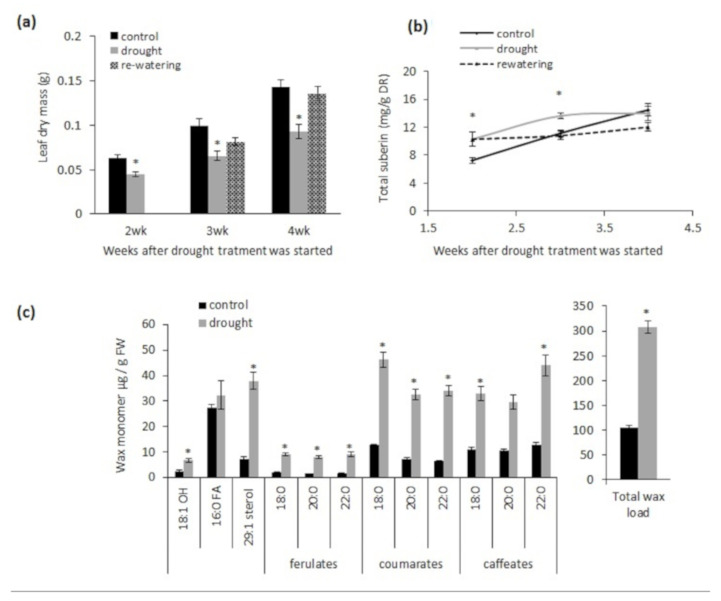
Comparison of leaf dry biomass, root suberin content, and root suberin-associated wax amounts in wild-type (Col-0) plants. (**a**) Leaf biomass, (**b**) root poly-aliphatic suberin at different time points under control, drought, and re-watering conditions, and (**c**) chloroform-extractable root wax amounts from 5-week-old tap root surfaces, 3 weeks drought stressed versus control. The drought treatment was at 5% (*v*/*v*) water level. In (**a**,**b**), plants were subjected to re-watering (20% water level) after two weeks of drought treatment (i.e., 3 weeks = 2 weeks drought + 1 week re-watering and 4 weeks = 2 weeks drought + 2 weeks re-watering). Data points shown are mean values in grams of leaf dry mass from 10 replicate plants ± SE (**a**), milligrams of total poly-aliphatic suberin per gram of delipidated dry residue from five replicate samples (a replicate consisted of roots from a pool of 4 plants) ± SE (**b**), and micrograms of total root wax per gram of fresh weight from 4–5 replicate samples (a replicate consisted of 10 tap roots) ± SE (**c**). Asterisks indicate *p* < 0.05 by Student’s *t*-test comparing the values of the control and treatment. 1-OH, primary fatty alcohol; FA = free fatty acid.

We next performed a time-course comparison of poly-aliphatic suberin production between drought-stressed and control (‘unstressed’) plants in the same time period. Our preliminary investigations revealed that introducing drought stress in sand-grown Arabidopsis when plants were younger than 2-weeks old (i.e., 2 weeks after sowing) resulted in high seedling-death rates. In addition, introducing drought stress for only one week did not result in a significant difference in suberin production between control and drought-stressed plants. However, when the length of the drought treatment was extended to 2 weeks, there was a significant difference in suberin production between control and drought-stressed plants. Therefore, the comparisons in suberin production and leaf biomass between control and drought-stressed plants were carried out in 4-week-old plants and older. Plants were 2-weeks old (after sowing) when the drought stress was introduced. Drought stress reduced the leaf biomass of wild-type plants after 2, 3, and 4 weeks (Figure 2a). Total suberin content after 2 and 3 weeks of drought stress was increased by 41% and 22%, respectively, relative to control (non-drought) conditions (Figure 2b). Control plants reached their highest suberin content 6 weeks after sowing, whereas plants under drought stress reached the highest suberin content 5 weeks after sowing (Figure 2b). However, despite the accelerated suberin production under drought, the total suberin content at maturity (6 weeks of age, including 4 weeks of drought stress) was not different than control conditions. In addition, no ectopic suberization was detected in the tap roots of plants exposed to drought stress (Figure S1). Plants re-watered after two weeks of drought reached the same biomass as the control plants (Figure 2a). There was no increase in total suberin content during the 2 weeks of re-watering (Figure 2b). Interestingly, 3 weeks after drought treatment, the total amount of root suberin-associated waxes was three times higher than that of control plants (Figure 2c). Except for 16:0 FA, all the characterized components of this fraction increased proportionally, indicating that water stress did not alter the composition of suberin-associated waxes.

### 2.2. Chronic Drought Stress Has a Similar Effect on Suberin-Deficient Mutants versus Wild-Type Plants

**Figure 3:**
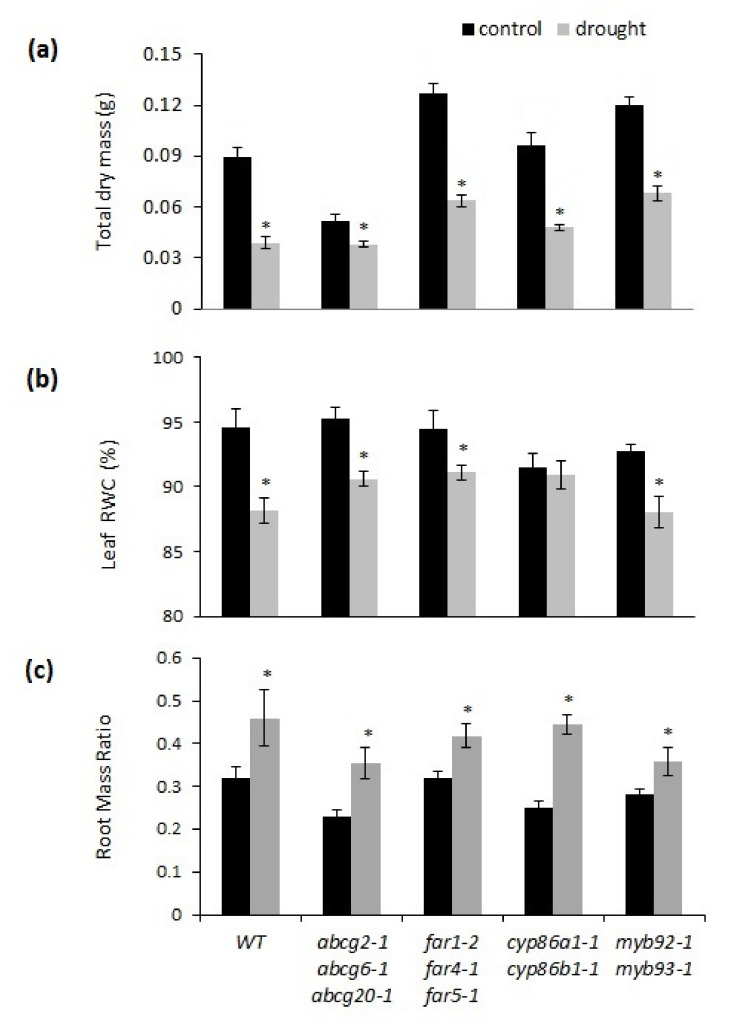
Comparison of drought stress responses between wild-type and suberin mutants. (**a**) Total plant dry mass, (**b**) leaf relative water content (RWC), (**c**) root mass ratio (ratio between root dry mass and total dry mass). Mean values from ten replicate plants ± SE. ANOVA results indicated significant genotype and treatment interactions for total plant dry mass at *p* < 0.01, and for RWC at *p* < 0.05 (Table S1). Asterisks indicate significant differences by Student’s *t*-test at *p* < 0.05 comparing control and treatment.

The chronic drought stress had a negative effect on the overall growth (total dry mass) of all genotypes investigated, but with some significant differences among them (Table S1 and Figure 3a). While the total dry mass was reduced by 56%, 50%, 50%, and 43% in the wild type, *far1-2 far4-1 far5-1*, *cyp86a1-1 cyp86b1-1*, and *myb92-1 myb93-1*, respectively, it was reduced by only 27% in *abcg2-1 abcg6-1 abcg20-1*, which had the lowest total dry mass under control conditions. Wilting symptoms were not displayed in any of the mutants, but compared to non-drought conditions, drought significantly reduced the leaf relative water content (RWC) in all genotypes except for *cyp86a1-1 cyp86b1-1* (Figure 3b). *Cyp86a1-1 cyp86b1-1* had the lowest average leaf RWC, indicating that it was the most-stressed genotype in terms of the water content in leaves, even under control conditions. All genotypes increased their biomass allocation to roots in response to drought stress with no significant interaction between drought and genotype (Figure 3c, Table S1).

### 2.3. Cyp86a1-1 cyp86b1-1 Has Reduced Total Suberin Content and Ultrastructural Defects

At the time this experiment was conducted, two of the Arabidopsis mutants selected for our study, namely *cyp86a1-1 cyp86b1-1* and *myb92-1 myb93-1*, had not been chemically and structurally characterized. Therefore, we investigated their suberin composition and lamellae structure. Of note, a recent study reported the suberin composition of *myb41 myb53 myb92 myb93*, a quadruple mutant that shows an overall ~ 78% decrease in suberin monomers compared to the wild type [30].

**Figure 4:**
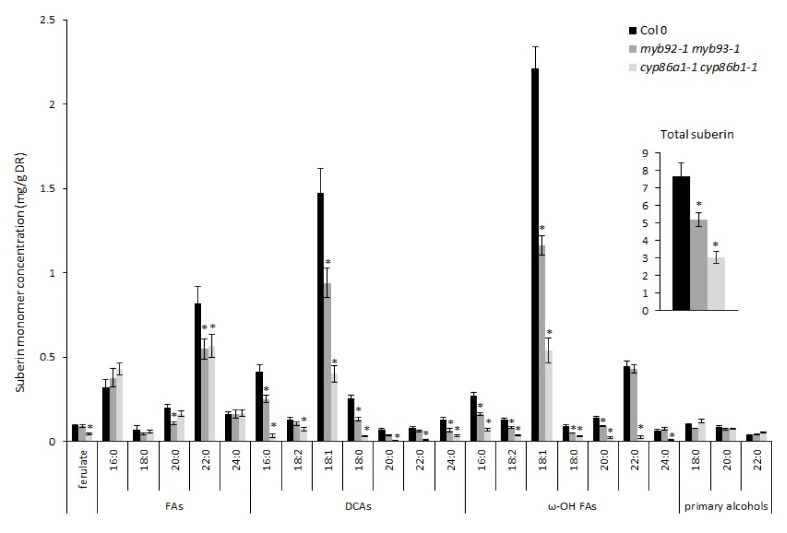
Comparison of root suberin monomer composition between the wild type (Col-0) and mutants *myb92-1 myb93-1* and *cyp86a1-1 cyp86b1-1*. Mean values are shown in milligrams of suberin monomer per gram of delipidated dry residue (DR) from 3–4 replicate samples ± SE. A replicate consisted of a pool of 4 plants at 4 weeks of age. Inset represents the total poly-aliphatic Scheme 0. FA, fatty acids; DCA, dicarboxylic acids; ω-OH FA, omega-hydroxy fatty acids. Asterisks indicate *p* < 0.05 by Student’s *t*-test comparing the values of the mutants to wild-type (Col 0)

Our preliminary results showed that compared to single mutants *myb92-1* and *myb93-1*, the double mutant *myb92-1 myb93-1* had a lower total suberin content (Figure S2). One-way ANOVA test results indicated significant differences in root suberin among these genotypes (*p* = 0.000). Compared to WT, *myb92-1*, *myb93-1*, and the double mutant *myb92-1 myb93-1* showed 22%, 11%, and 40% reduction in the total suberin content, respectively (Figure S2). Under non-drought conditions, the *cyp86a1-1 cyp86b1-1* double mutant showed a 60% reduction in total suberin content compared to the wild type (Figure 4). The decrease was associated with significant reductions in ferulate, 22:0 FA, and all DCAs and ω-OH FAs. Similarly, in the absence of stress, the total suberin content in *myb92-1 myb93-1* was 33% lower than that of the wild type in this second experiment, showing lower levels of 20:0 and 22:0 FAs, 16:0, 18:1, 18:0, and 24:0 DCAs, and 16:0, 18:2, 18:1, 18:0, and 20:0 ω-OH FAs (Figure 4).

**Figure 5:**
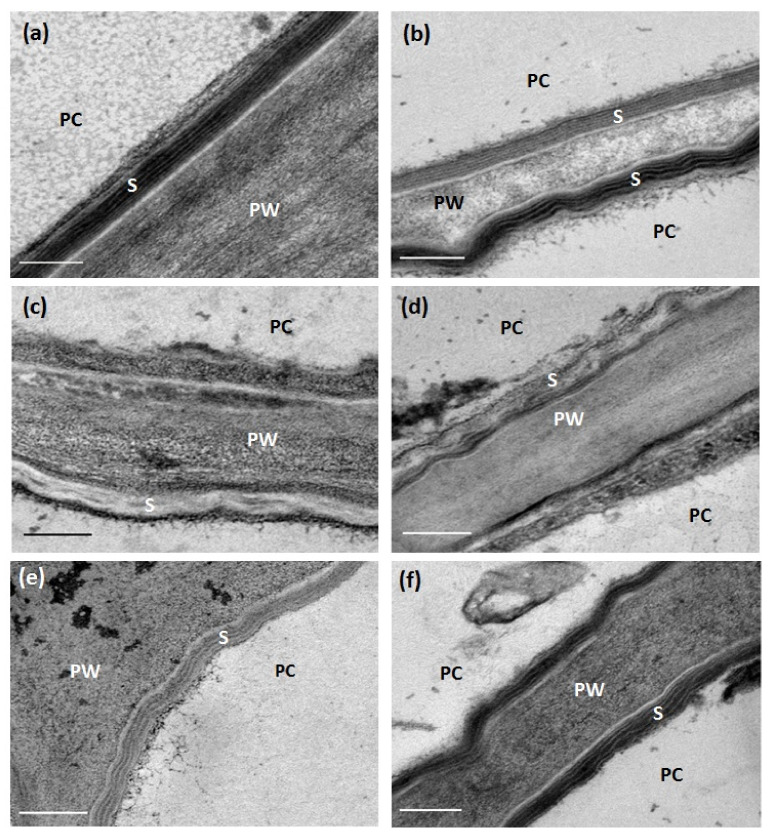
Transmission electron microscope images of *A. thaliana* root sections of the wild type and mutants showing suberin lamellae in the root periderm. Sections were taken from tap roots of 4-week-old wild-type, *cyp86a1-1 cyp86b1-1*, and *myb92-1 myb93-1* plants grown under control and drought-stress conditions. (**a**) Wild type, control; (**b**) wild type, drought; (**c**) *cyp86a1-1 cyp86b1-1*, control; (**d**) *cyp86a1-1 cyp86b1-1*, drought; (**e**) *myb92-1 myb93-1*, control; (**f**) *myb92-1 myb93-1*, drought. S, suberin lamellae; PW, primary cell wall; PC, periderm cell. Scale bar = 200 nm.

We then analyzed the ultrastructure of root periderm suberin of non-drought-stressed plants by transmission electron microscopy. In contrast to the suberin lamellae of wild-type roots, *cyp86a1-1 cyp86b1-1* did not contain the characteristic light and dark banding pattern (Figure 5a,c). On the other hand, the *myb92-1 myb93-1* root periderm had a suberin lamellae structure similar to that of the wild type (Figure 5a,e).

### 2.4. Chronic Drought Stress Increases Root Suberin Content but Does Not Alter Its Lamellae Structure

**Figure 6:**
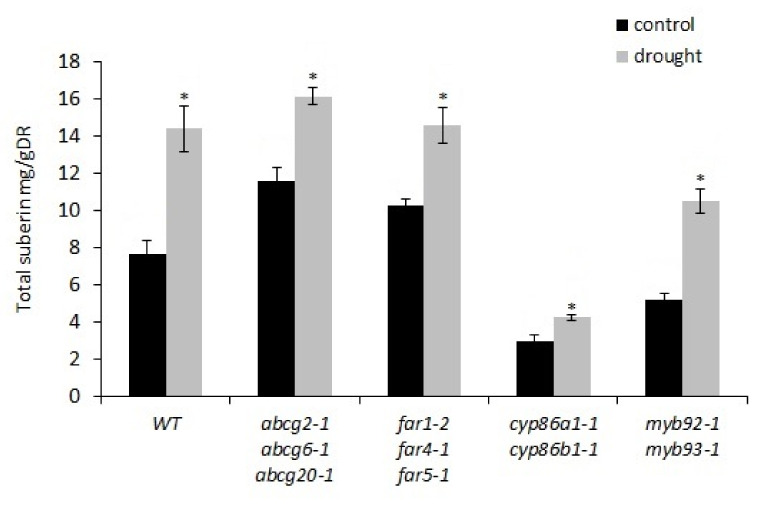
Total poly-aliphatic suberin in roots of wild-type and suberin mutants. Mean values of total suberin are given in milligrams of suberin per gram of delipidated dry residue (DR) from 3–4 replicate samples ± SE. Each replicate consisted of roots from four pooled plants at 4-weeks old. ANOVA results indicated significant interactions between genotype and treatment effects (Table S1). Asterisks indicate significant differences by Student’s *t*-test at *p* < 0.05 comparing control and treatment.

Drought increased the root suberin content in all genotypes (Table S1). The level of induction was similar in the wild type, *abcg2-1 abcg6-1 abcg20-1*, and *far1-2 far4-1 far5-1* (Figure 6). Compared to the control condition, the wild type had an 89% increase in total suberin content under drought stress (Figure 6), with increases in all monomer classes (Figure S3). Drought doubled the amount of suberin in *myb92-1 myb93-1* (Figure 6), which was associated with significant increases in FAs, DCAs, and ω-OH FAs (Figure S3). *Cyp86a1-1 cyp86b1-1* recorded the lowest (42%) increase in total suberin content after drought stress, resulting from increases in FAs, DCAs, and primary fatty alcohols (Figure S3). Nevertheless, in the wild type, *cyp86a1-1 cyp86b1-1*, and *myb92-1 myb93-1*, the increased amount of suberin due to drought stress did not change the lamellae structure, which in all cases remained the same as in their respective control conditions (Figure 5).

### 2.5. Suberin Deficiency and Aberrant Lamellae Structure Correlates with Increased Water Loss in Root Periderm

**Figure 7:**
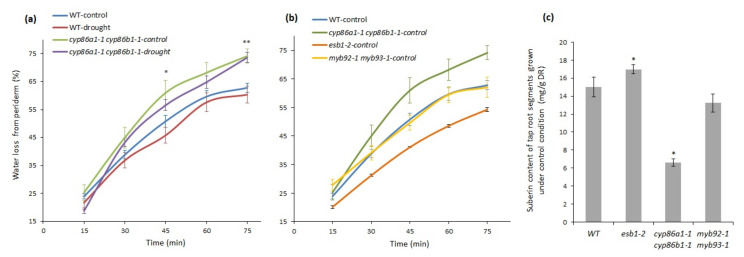
Comparison of amount of water loss from root periderm segments in the wild type and mutants altered in total poly-aliphatic suberin. (**a**) Amount of water loss in the wild type and *cyp86a1-1 cyp86b1-1* under control and drought conditions. (**b**) Amount of water loss in wild-type, *cyp86a1-1 cyp86b1-1, esb1-2*, and *myb92-1 myb93-1* tap root periderm segments under control condition (*n* = 5, with each replicate representing three root periderm segments from different plants). Values are the mean ± SD of five replicate samples. Significant differences in (**a**) between wild-type drought and *cyp86a1-1 cyp86b1-1-*drought conditions are shown by asterisks (* *p* < 0.05 and ** *p* < 0.01 calculated by Student’s *t*-test). (**c**) Total suberin content from wild-type, *cyp86a1-1 cyp86b1-1*, *esb1-2*, and *myb92-1 myb93-1* periderm segments. (*n* = 4–5, with each replicate representing roots from four pooled plants at 4 weeks of age). Asterisks in (**c**) indicate significant differences by Student’s *t*-test at *p* ≤ 0.05 comparing wild-type and mutants.

Under control conditions, *cyp86a1-1 cyp86b1-1* recorded the lowest average RWC, indicating that it was the most-stressed mutant of all the genotypes tested. This prompted us to examine whether the root periderm of this double mutant is more permeable to water than the periderm of other genotypes, as it has reduced total suberin content, altered monomer composition, and ultrastructural defects. We compared the amount of water loss in mature root periderm segments of *cyp86a1-1 cyp86b1-1* with that of the wild type. Water loss from root periderm of *cyp86a1-1 cyp86b1-1* was 15% higher than from root periderm of the wild type, both under control and drought conditions (Figure 7a). Genotype effect was significant after 45 and 75 min (*p* < 0.01, Student’s *t*-test). However, in both wild-type and *cyp86a1-1 cyp86b1-1* plants, there was no significant difference in root water loss between the control and drought treatment (Figure 7a). Furthermore, except for a marginal increase in *cyp86a1-1 cyp86b1-1*, there was no change in periderm suberin content under drought stress in all other genotypes (data not shown). Therefore, in the next experiment we used plants that were grown only under control (non-drought) conditions to investigate if total suberin content helps to reduce water loss through roots (Figure 7b).

An additional mutant, *esb1-2*, which has unaffected suberin lamellae [37] and a higher root poly-aliphatic suberin content than the wild type [28,37], was included in this experiment. *Esb1* is defective in the formation of Casparian bands in the endodermis. However, in this mutant, ectopic deposition of suberin lamellae has been observed between the plasma membrane and the cell wall in both endodermal cells close to the root tip and endodermal passage cells [37]. Our analysis also showed increased suberin content in the *esb1-2* root periderm (Figure 7c), which has not been previously characterized in this mutant. Water loss through the *esb1-2* root periderm under control conditions was 15% lower than that of the wild type at 75 min (Figure 7b). Suberin content in periderm segments was highest in *esb1-2* and lowest in *cyp86a1-1 cyp86b1-1* (Figure 7c), while wild-type and *myb92-1 myb93-1* periderm segments recorded intermediate levels of suberin (Figure 7c). These results suggest an inverse relationship between periderm suberin content and water loss through the periderm. A separate experiment showed that under control conditions, total suberin in *esb1-2* had 30% more suberin than the wild type (Figure S4), and leaf RWC values in control and drought-stressed *esb1-2* were the same as wild-type control plants (Figure S4). The drought stress did not have an impact on the water status (RWC) of *esb1-2* under drought stress, possibly due to reduced water loss through the periderm. However, there was no increase in total suberin content by drought stress, likely because the suberin biosynthetic capacity of periderm cells was maximized in *esb1-2* under both the control and drought conditions (Figure S4). Taken together, our results provide evidence that Arabidopsis periderm suberization is inversely correlated with its permeability to water.

### 2.6. Salt Stress Induced Suberin Biosynthesis in Roots of Wild-Type Plants

**Figure 8:**
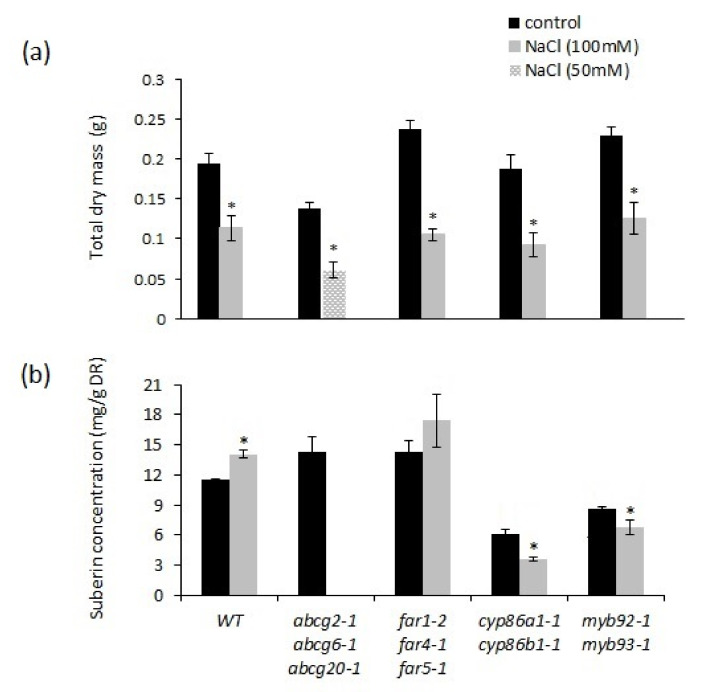
Comparison of total dry mass and total suberin content in wild-type and suberin mutants under control and after treatment with 50 mM or 100 mM NaCl for 2 weeks. Wild-type, *far1-2 far4-1 far5-1*, *cyp86a1-1 cyp86b1-1*, and *myb92-1 myb93-1* plants were exposed to 100 mM NaCl and *abcg2-1 abcg6-1 abcg20-1* was exposed to 50 mM NaCl. (**a**) Total dry mass; (**b**) total poly-aliphatic suberin. *Abcg2-1 abcg6-1 abcg20-1* NaCl treatment data are not included in Figure (**b**), as sufficient root biomass was not present at harvesting. *Abcg2-1 abcg6-1 abcg20-1* mutant was also excluded from the statistical analysis. Mean values in dry mass are given in grams from ten replicate samples ± SE. Each replicate consisted of one individual plant. Mean values of suberin contents are given in milligrams of suberin per gram of delipidated dry residue (DR) from 3–4 replicate samples ± SE. Each replicate consisted of roots from 4–5 separate plants. ANOVA results indicated significant interactions between genotype and treatment effects in root suberin data at *p* < 0.01 (Table S2). Asterisks indicate significant differences by Student’s *t*-test at *p* < 0.05 comparing control and NaCl treatment.

Next, we investigated how mutants with defects in root suberization respond to NaCl treatment. Growth of all genotypes was reduced by 100 mM NaCl treatment (Figure 8a and Figure S5). In a preliminary experiment, there was 95% mortality of the *abcg2-1 abcg6-1 abcg20-1* mutant at 100 mM NaCl. Therefore, the *abcg2-1 abcg6-1 abcg20-1* plants were exposed to 50 mM NaCl to compare the severity of stress, but these data were not included in the statistical analysis. The effect of NaCl treatment on total biomass did not differ among genotypes (Figure 8a; Table S2), except for the high mortality of the *abcg2-1 abcg6-1 abcg20-1* mutant. The salt stress reduced the growth of *abcg2-1 abcg6-1 abcg20-1* even at 50 mM NaCl, and the root biomass obtained was not sufficient to analyze poly-aliphatic suberin in these salt-stressed plants. Therefore, suberin data for *abcg2-1 abcg6-1 abcg20-1* under salt stress are not included in Figure 8b and Figure S6a. NaCl treatment caused a 22% increase in total suberin in wild-type plants (Figure 8b and Table S2). For *cyp86a1-1 cyp86b1-1* and *myb92-1 myb93-1*, the two genotypes with the lowest total poly-aliphatic suberin under control conditions, salt stress resulted in root poly-aliphatic suberin decreases of 41% and 21%, respectively. The salt treatment did not affect total poly-aliphatic suberin content in *far1-2 far4-1 far5-1* (Figure 8b). DCAs increased and FAs were decreased both in the wild type and in *far1-2 far4-1 far5-1*. In contrast, the NaCl treatment caused reductions in the most-abundant monomer groups (FAs, DCAs, and ω-OH FAs) in *cyp86a1-1 cyp86b1-1* (Figure S6c) and in FAs of *myb92-1 myb93-1* (Figure S6d). Primary alcohols were not affected by NaCl treatment in any of the genotypes (Figure S6). There was no significant increase in total root suberin-associated waxes by the NaCl treatment, but significant increases were observed in 18:0 ferulate and 20:0 and 22:0 coumarates (Figure S7).

### 2.7. NaCl Treatment Causes Ion Imbalances in Mutants with Altered Suberin Composition and Structure

**Figure 9:**
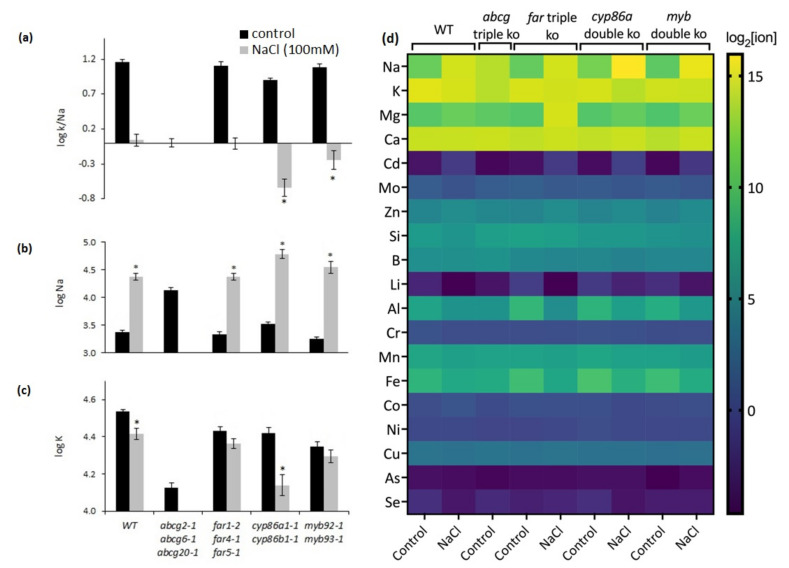
Analysis of elements in leaf tissues of wild type and mutants under control and NaCl treatment. (**a**) log K/Na, (**b**) log Na, (**c**) log K. (**d**) Analysis of elements (left) in leaf tissues. The heat map represents the abundance of each analyzed element (Table S4) as log_2_ of each element concentration given in parts per million (ppm) under control (0 mM NaCl) and 100 mM NaCl treatments. For each element, data are derived from 4–5 plants. *Abcg2-1 abcg6-1 abcg20-1* NaCl treatment data are not included in the figure and statistical analysis due to salt hypersensitivity. Mean values in (**a**–**c**) are from 4 replicate samples ± SE. Each replicate represents a pool of 4–5 leaves from an individual plant. In (**a**), Asterisks indicate the differences at *p* < 0.05 by Student’s *t*-test comparing NaCl-treated wild-type and NaCl-treated mutant plants. In (**b**,**c**), asterisks indicate the differences at *p* < 0.05 by Student’s *t*-test comparing the control and NaCl treatment. ANOVA results indicated significant interactions between genotype and treatment effects in leaf Na at *p* < 0.05 and leaf K at *p* < 0.01 (Table S2).

The potential effect of the different responses of root suberin content on leaf elemental composition between the different genotypes was examined by Principal Component Analysis (PCA) (Table S4; Figure S8). The PCA showed a clear separation of the *abcg2-1 abcg6-1 abcg20-1* triple mutant from the other genotypes with respect to elemental content under control conditions, the only condition shown for this mutant since it did not survive the NaCl treatment (Figure S8a). *Cyp86a1-1 cyp86b1* was separated in the other direction compared to that of *abcg2-1 abcg6-1 abcg20-1* (Figure S8a). After NaCl treatment, the other genotypes had remarkably similar elemental compositions, with *far1-2 far4-1 far5-1* showing the widest scatter (Figure S8b). Except for *abcg2-1 abcg6-1 abcg20-1*, NaCl treatment decreased the leaf K/Na ratio in all genotypes, with the largest decreases in *cyp86a1-1 cyp86b1-1* and *myb92-1 myb93-1*, in which Na concentrations of the stressed plants were higher than the K concentrations (Figure 9a–c). *Abcg2-1 abcg6-1 abcg20-1* did not survive the stress caused by NaCl addition, but it had a similar leaf K/Na ratio under control conditions as that of the wild type and the *far1-2 far4-1 far5-1* mutant under NaCl treatment (Figure 9a). The changes in the K/Na ratio were mostly caused by increased Na concentration, except for *cyp86a1-1 cyp86b1-1*, which also had a lower K concentration under salt stress (Figure 9). Analyses of leaf micronutrient contents showed differences among genotypes for only boron (B), silicon (Si), manganese (Mn), and copper (Cu) (*p* < 0.01, Kruskal–Wallis) (Figure 9d and Table S5). Taken together, these results highlight that among the surviving genotypes after 100 mM NaCl treatment, *cyp86a1-1 cyp86b1-1* presented the highest ion imbalance.

## 3. Discussion

In this study, we report changes in Arabidopsis root suberin accumulation in response to chronic water deficiency and high NaCl in wild-type plants. In addition, to better understand how altered suberin amount, composition, and lamellae structure in roots regulate water and ion transport, the stress responses of selected mutants with different degrees of suberin deficiency were investigated. The results show that (1) chronic drought stress increases the rate of suberin biosynthesis and the total root suberin-associated wax content in wild-type roots; (2) suberin production exhibits plasticity under different soil water contents; (3) chronic drought stress, but not increased NaCl, induces suberin in all tested genotypes; and (4) mutants with suberin deficiency and distorted lamellae experienced increased water loss through the root periderm as well as ion imbalances upon NaCl treatment relative to the wild type.

### 3.1. Wild-Type Roots Show Plasticity in Suberin Production in Response to Different Water Levels

Previous studies have examined changes in developmental suberin deposition under non-stress conditions [9,11] in Arabidopsis and under osmotic stress conditions in barley [15]. Here we investigated the pattern of Arabidopsis root suberin deposition over a time-course of development under both non-drought (‘non-stress’) and drought stress conditions. We observed that drought accelerates suberin deposition in all tested genotypes, indicating that drought induces suberin biosynthesis. When the drought stress imposed on wild-type plants was removed (i.e., plants were re-watered to the control condition moisture level), the effect on suberin disappeared. Our results provide evidence for the controlled regulation of suberin biosynthesis at different levels of soil water, similar to the plasticity previously reported in response to different nutrient availability [24]. Drought stress causes induction of ABA in roots [38]. The higher rate of suberin biosynthesis is likely one of the drought responses regulated by abscisic acid (ABA), which has been shown to be involved in regulating suberin deposition [24]. The deposition of root suberin-associated waxes in response to drought stress has not been previously reported. We observed increases in suberin-associated waxes due to drought in the wild type, which suggests a role in the protection against water loss and minimizing the risk of cavitation in xylem conduits [39,40].

### 3.2. Root Suberin Modulates Water Loss through the Periderm

We tested various mutants differentially altered solely in suberin composition or altered in both composition and lamellae structure to evaluate whether the analyzed mutants have different vulnerabilities to stresses. We observed a 60% decrease in root suberin content in the double *cyp86a1-1 cyp86b1-1* mutant, which also presented a loss of lamellae ultrastructure. In contrast, a 33% decrease in root suberin content in the *myb92-1 myb93-1* double mutant was not associated with a deformed lamellae structure. These two mutants had differences in their monomer compositions, but our analysis cannot determine which specific type(s) of monomers, if any, are critical to forming the lamellar structure observed by TEM. On the other hand, the *abcg2-1 abcg6-1 abcg20-1* mutant has many defects in suberin composition, but its total suberin content is higher than that of the wild type, and the mutant fails to form suberin lamellae [32]. The *far1-2 far4-1 far5-1* mutant shows changes in suberin monomer composition, specifically near the elimination of primary fatty alcohols, but it has normal total suberin content and lamellae structure [10]. Thus, it is possible that an adequate balance of certain monomer types, together with a minimal total amount of poly-aliphatic suberin, is needed to achieve such ultrastructural organization. Nevertheless, our results did indicate that the native ultrastructure of suberin is functionally critical in reducing water loss under drought stress. Similarly, Kreszies et al. [41] also emphasized that monomer arrangements and suberin ultrastructure play an important role in the proper functioning of the suberin polymer. We must also consider the possibility that roots of plants exposed to drought conditions can present ectopic suberization, thereby also increasing the total load of suberin [24]. In addition, the *MYB41* and *GPAT5* promoters drive the expression of reporter genes in cortical cells under ABA and NaCl stresses [24,42]. However, these studies were conducted with very young roots in contrast to our experiments, where suberin would be mainly deposited in the periderm and the endodermis and cortex are already sloughed off (Figure S1).

The root periderm acts as the first line of defense against the underground environment. Suberin is a protective biopolymer found in the periderm and endodermis of roots, yet its specific contribution to the stress response function of the root periderm has not been elucidated. Under control conditions, the *cyp86a1-1 cyp86b1-1* double mutant presented the lowest leaf relative water content, implying it is the most water-stressed genotype. This mutant showed a high rate of water loss through the root periderm and severely decreased root suberin content with a distorted ultrastructure. Conversely, *esb1-2*, a mutant with high levels of poly-aliphatic suberin ectopically deposited in roots [28] and a lamellae structure similar to the wild type [37], had a low rate of water loss through the root periderm. Thus, suberin composition and ultrastructure play a role in preventing uncontrolled water loss from the root periderm. Similarly, Arabidopsis mutants affected in the phenylpropanoid pathway present a dysfunctional periderm with lower suberin content than the wild type as well as reduced salt tolerance [43]. A lower rate of water loss helps to improve water-use efficiency (WUE) under drought stress [44]. Enhanced suberin deposition in *esb1-2* was associated with higher WUE [28,37] that appears to be a consequence of reduced transpirational losses through reduced stomatal apertures in leaves [45].

The drought stress response observed in *far1-2 far2-1 far5-1* was similar to the wild type, which indicates that primary alcohols that are part of the suberin polyester and root suberin-associated waxes (where they provide the alkyl moiety of alkyl hydroxycinnamates) do not play a major role in resistance to chronic drought stress. Vishwanath et al. [10] showed that suberin-associated waxes in the root periderm of 4-week-old *far1-2 far2-1 far5-1* are more affected than the suberin polymer. Despite the defects in root suberin-associated waxes, the physiological parameters tested in this study did not indicate any chronic drought susceptibility in *far1-2 far2-1 far5-1*.

### 3.3. Mutants Defective in Suberin Composition and Lamellae Structure Show Higher Sensitivity to NaCl Treatment

Recently, the effects of NaCl on the hydroponically grown wild type and the *cyp86a1* mutant were compared [27]. *Cyp86a1* exhibited a salt-sensitive phenotype, with 5-week-old hydroponic-grown *cyp86a1* plants displaying more withered leaves and down-curling of leaf tips after a 50 mM NaCl treatment [27]. Additionally, *cyp86a1* leaf membranes suffered more serious injuries by NaCl treatment [27]. Similar to the findings of that study, we observed increases in root suberin deposition in the wild type but not in the *cyp86a1 cyp86b1* double mutant. Additionally, there was an increase in Na accumulation and a reduction in K in shoots in the *cyp86a1-1* single mutant [27]. *Abcg2-1 abcg6-1 abcg20-1* recorded a very high Na content and a very low K content even under control conditions, indicating increased movement of Na to the shoot, while the *far1-2 far4-1 far5-1* mutant was not affected by NaCl treatment. Thus, the ability of a plant to tolerate salinity can depend on how much it limits Na transport to the shoot, while maintaining K content [46]. Therefore, our results provide evidence that both *cyp86a1-1 cyp86b1-1* and *abcg2-1 abcg6-1 abcg20-1* mutants have reduced tolerance to salt stress due to high Na and low K in shoot tissues. In contrast, increased suberin in the *esb1-2* mutant reduced solute leakage in roots [28]. Krishnamurthy et al. [19] also detected a similar relationship among rice cultivars that varied in tolerance to salinity. The salt-tolerant cultivar Pokkali has more suberin in its roots than the salt-sensitive cultivar IR20. The exposure of both cultivars to 100 mM NaCl resulted in the deposition of additional suberin, which reduced the accumulation of Na in the shoot and improved the survival of plants when subjected to 200 mM NaCl [19]. High Na is known to cause increases in K efflux [27,47]. Maintaining a high K/Na ratio, either by the retention of K or by preventing Na from accumulating in leaves is essential for salt tolerance [48]. A decreased K concentration under salt stress in Arabidopsis has been reported to be mainly due to NaCl-stimulated K efflux in roots [27]. Additionally, membrane depolarization due to Na entry leads to the efflux of K, and additionally, a high NaCl concentration impairs the integrity of the plasma membrane, resulting in a release of cellular solutes including K [49,50,51]. Therefore, we speculate that *abcg2-1 abcg6-1 abcg20-1* suffered from ion imbalance due to uncontrolled uptake of Na, which resulted in high mortality by NaCl treatment. The *cyp86a1-1 cyp86b1-1* and *myb92-1 myb93-1* mutants recorded significantly decreased leaf K/Na ratios relative to the wild type, but these two mutants appeared to have suffered less from ion imbalance than that of *abcg2-1 abcg6-1 abcg20-1*.

In conclusion, our results indicate that the chemical composition and ultrastructure of suberin help to prevent water loss and limit uncontrolled Na uptake under high-salinity conditions in Arabidopsis. The results also indicate that suberin synthesis shows plasticity at different water levels and prevents root dehydration under chronic drought stress, which is important to maintain water balance in a plant. Additionally, our study showed that under drought stress, *cyp86a1-1 cyp86b1-1* and *myb92-1 myb93-1* had increased suberin, while these genotypes had decreased suberin under NaCl stress, indicating different suberin regulatory mechanisms in different stresses. These findings provide evidence for the regulated deposition of suberin in the root periderm as a mechanism to protect against abiotic stresses and contribute to future research targeting enhanced stress resistance in crops.

## 4. Materials and Methods

### 4.1. Plant Growth Conditions and Drought and Salt Assays

*Arabidopsis thaliana* wild type (Col-0 ecotype) and double and triple T-DNA insertion lines of suberin mutants were either generated by the authors or obtained from the Arabidopsis Biological Resource Center (ABRC). Homozygous T-DNA insertion lines were confirmed by PCR genotyping using primers listed in Table S3. Seeds of the wild type and mutants were surface sterilized and stratified for 3 to 4 days at 4 °C. For growth, 250 mL plastic cups with mesh-covered holes for drainage were filled with commercial play sand (2 mm sieved; pH 6.3; KING’s brand, Brantford, ON, Canada). The sand allowed us to precisely control the water content gravimetrically and harvest roots without damage. Initially, 50 mL of 20–20–20 nitrogen–phosphorus–potassium fertilizer (1 g/L solution) (Plant-Prod, Brampton, ON, Canada) was added to each pot and then replenished biweekly with 3 mL of fertilizer solution. The amount of water required for 20% (*v*/*v*) was calculated based on the volume of the cup. Thirty pots from each genotype were kept at 20% (*v*/*v*) water content by adding the required amount of water daily. The growth chamber conditions were 21–22 °C, 40–60% humidity, and long-day conditions (16 h light/8 h dark cycle). Two to three seeds were sown initially and kept covered with transparent plastic domes for one week and thinned out to one healthy seedling after one week. To induce the drought stress, watering was withdrawn in 20 pots from each genotype on the 10th day after sowing. Three days after water withdrawal, the soil moisture level reached 5% (*v*/*v*) and was maintained at that level throughout the experiment for drought-stressed plants. To analyze the regulation of suberin production under re-watering conditions, two weeks after drought stress was introduced, 10 replicate pots were watered again to reach 20% (*v*/*v*). For the salt-stress assay, 20 pots were maintained at 20% water. To induce the salt stress in wild-type and mutant plants, 0.1 g of NaCl was added to 10 replicate pots of each genotype for three consecutive days starting on the 10th day after sowing. The NaCl concentration in the soil solution reached 100 mM on the 14th day (2 weeks after sowing). Since preliminary studies revealed a high rate of salt-induced mortality in the *abcg2-1 abcg6-1 abcg20-1* mutant, 50 mM NaCl was instead used for this mutant by adding 0.15 g of NaCl on the 10th day after sowing of that mutant.

### 4.2. Plant Trait Measurements

At four weeks of age, a fully developed young leaf from each plant was collected for drought-stressed and control treatments to determine the leaf relative water content (RWC). The leaves were stored in a re-sealable plastic zipper bag, and the fresh masses (FM) were determined immediately. These leaves were then hydrated overnight by placing them between moist paper towels for 24 h, after which the saturated mass of the fully turgid leaves (SM) was measured [52]. Leaves were then dried for 48 h at 80 °C, and their dry mass (DM) was recorded. The leaf RWC was calculated as the ratio (FM–DM)/(SM–DM) [53]. For root suberin analysis, the root system was carefully harvested by rinsing off the sand and gently drying with paper wipes. For total biomass analysis, the above-ground plant parts (inflorescence, cauline leaves, and rosette leaves) and roots were dried separately in an oven for 48 h at 80 °C in paper bags.

### 4.3. Root Wax Extraction and Analysis

Five-week-old wild-type tap roots were used in this study because it has been found that suberin-associated wax deposition mostly takes place after 4 weeks [54]. Each treatment (control or three weeks of drought stress) was represented by 4–5 replicates, and each replicate consisted of 10 tap roots. Root fresh weights were recorded, and the roots were dipped in chloroform for 1 min. The solution contained 5 μg each of *n*-tetracosane (24:0 alkane), 1-pentadecanol (15:0-OH), heptadecanoic acid (17:0), and tridecyl (13:0)-ferulate, which served as internal standards. Extracts were evaporated under nitrogen and derivatized with 100 μL of *N*,*O*-*bis*-(trimethylsilyl)-trifluoroacetamide (BSTFA) plus 100 μL of pyridine at 110 °C. The derivatized samples were allowed to cool and the solvent evaporated under nitrogen. The samples were re-suspended in heptane:toluene (1:1 *v*/*v*) for analysis by gas chromatography (GC) following the method described in [54].

### 4.4. Root Suberin Analysis

Roots were immersed in 4 mL of hot isopropanol and incubated for 30 min at 85 °C. The samples were delipidated for 24 h each with chloroform:methanol (2:1, *v*/*v*), chloroform:methanol (1:1, *v*/*v*), chloroform:methanol (1:2, *v*/*v*), and 100% methanol as described in [10]. Sodium methoxide-catalyzed depolymerization and GC-mass spectrometry (MS) and GC-flame ionization detector (FID) analyses were performed following the protocol detailed in [55].

### 4.5. Transmission Electron Microscopy (TEM)

Microwave processing of the samples was adapted from [56,57]. Samples were processed using a Pelco Biowave 34700 microwave (TedPella, Redding, CA, USA). All microwave steps were performed under vacuum (>20 mm Hg), except dehydration and polymerization. One-mm-long tap root segments, which were taken immediately below the hypocotyl junction (Figure S9) from 4-week-old wild-type and mutant plants grown in 20% moisture (control) or 5% moisture (drought) levels, were submerged in 0.1 M Na cacodylate buffer (pH 7.2). The samples were transferred to 1.5 mL microcentrifuge tubes containing 600 μL of 2.4% glutaraldehyde and 2.0% paraformaldehyde in 0.1 M sodium cacodylate buffer (pH 7.2), de-gassed under vacuum (>20 mm Hg) for 20 min, and then put in the microwave, held under vacuum for 1 min, microwaved at 250 W with a 37 °C restrictive temperature for 40 s at 100% power, and held under vacuum for another 3 min. The samples were rinsed with sodium cacodylate buffer and then washed twice in the same buffer in the microwave under vacuum (>20 mm Hg) at 450 W at ≤40 °C, and then held under vacuum for 1 min 40 s at 100% power. The samples were post-fixed in 1% OsO_4_ in 0.1 M sodium cacodylate buffer in the microwave under vacuum (>20 mm Hg) at 450 W at ≤37 °C, held under vacuum for 1 min, 40 s at 100% power, and held under vacuum for another 3 min. Samples were rinsed with water and then placed in the microwave under vacuum (>20 mm Hg) at 250 W at ≤40 °C for 1 min 40 s at 100% power. Samples were then dehydrated via an ethanol series of 30, 50, 70, 80, 90, 95%, and twice with 100%, followed by two times in acetone. At each dehydration step, the samples were microwaved at 250 W at ≤37 °C for 40 s at 100% power. Infiltration was performed three times in Spurr low-viscosity resin (Electron Microscopy Sciences, Hatfield, PA, USA) in the microwave at 450 W at ≤43 °C for 2 min at 100% power. Samples were polymerized in capsules in the microwave at 750 W at ≤60 °C for 19 min at 100% power, at ≤70 °C for 12 min at 100% power, at ≤80 °C for 12 min at 100% power, and at ≤100 °C for 45 min at 100% power. Embedded tissues were sectioned to 100 nm thickness using a diamond knife and a Reichert Ultracut E ultramicrotome (Leica Microsystems, Vienna, Austria) and collected on copper grids that were coated with formvar and carbon (Electron Microscopy Sciences, Hatfield, PA, USA). Grids were treated with 10% hydrogen peroxide for 10 min and stained with 10% uranyl acetate in methanol for 8 min, and then Reynold’s lead citrate for 10 min [58]. The samples were analyzed with a Hitachi H-7000 TEM (Hitachi, Tokyo, Japan) equipped with an ORIUS SC200 digital camera using Digital Micrograph software version 1.8.3 (Gatan Inc., Pleasanton, CA, USA). Images were processed with Adobe Photoshop Version 7.0.

### 4.6. Measurement of Root Periderm Segments for Water Loss

The method described in [59] was used to measure water loss from the root periderm. An additional mutant, *esb1-2*, was used in this experiment [37]. For each genotype, weights were taken from a pool of three root periderm segments at the same developmental stage. A ~1 cm tap root segment at the base of the root system (Figure S9) was excised from individual 4-week-old plants (2 weeks after drought stress for drought treatment samples). A thin layer of Paraplast wax was applied to the cut surfaces to stop water loss through cut sites, and the weight was recorded immediately. The root segments were then placed on a bench and weights were recorded at 15 min intervals at room temperature (about 25 °C). The percent of water loss relative to the initial weight was recorded. The loss of fresh mass was calculated as the percentage of the initial mass.

### 4.7. Tissue Elemental Analysis

Leaf tissue samples were oven dried at 85 °C for 48 h in 7 mL perfluoroalkoxy (PFA) vials until the sample weight reached a constant weight such that the samples were completely dehydrated. The dried leaves were ground to a fine powder using a mortar and pestle, and approximately 10 mg of each sample was weighed in a PFA vial, lightly closed, and digested with 0.5 mL of 15 M HNO_3_ on a hotplate at 110 °C for 8 h. The solution was diluted into deionized water gravimetrically to 10 g and analyzed by microwave plasma-mass spectrometry (MP-MS) (Agilent 4200 MPES) and inductively coupled plasma-mass spectrometry (ICP-MS) (Agilent 8800QQQ Triple Quadrupole ICP-MS). The elements Ca, K, Mg, and Na were measured by MP-ES, and the elements Li, B, Al, Si, Cr, Mn, Fe, Co, Ni, Cu, Zn, As, Se, Mo, and Cd were measured by ICP-MS. Reproducibility due to sample heterogeneity and measurement uncertainty was evaluated using 10 mg replicate portions corresponding to the same homogenized plant specimen.

### 4.8. Statistical Analysis

Statistical analyses were performed using SYSTAT Version 13 (Systat Software Inc., Chicago, IL, USA). Prior to analyses, if necessary, log transformation of data was carried out to attain a normal distribution. To evaluate whether drought or NaCl treatment had any effect on the measured traits, ANOVA tests were performed. Significant interactions between treatment (drought or NaCl) and genotypes enabled us to identify the percent changes in tested parameters among different genotypes. The Kruskal–Wallis test was conducted when assumptions of normality were not met. Student’s *t*-test was conducted to determine if there was a significant difference between the means of two groups (either between control and treatment or wild type and mutant). Principal Component Analysis (PCA) was carried out to investigate the segregation of the leaf ionomic phenotype of the wild type and mutants using data obtained by ICP-MS.

## Data Availability

Data is contained within the article or Supplementary Material.

## Supplementary Materials

The following are available online at https://www.mdpi.com/article/10.3390/metabo11110735/s1, Figure S1: Microscopic view of suberin distribution in wild-type Arabidopsis roots under drought conditions, Figure S2: Total poly-aliphatic suberin in roots of wild-type, *myb92-1*, *myb93-1* and *myb92-1 myb93-1*, Figure S3: Root suberin composition under control and drought stress in wild-type and suberin mutants, Figure S4: Comparison of root suberin and leaf relative water content (RWC) in wild-type and *esb1-2* under control and drought conditions, Figure S5: Comparison of plant sizes of control (0 mM NaCl) and after treatment with 50 mM or 100 mM NaCl for 2 weeks, Figure S6: Comparison of root poly-aliphatic suberin composition in mutants and wild-type under control and NaCl treatments, Figure S7: Comparison of root suberin-associated wax composition in wild-type plants under control conditions and after 3 weeks of 100 mM NaCl treatment, Figure S8: Segregation of leaf ionomic phenotypes in wild-type and mutants by Principal Component Analysis (PCA), Figure S9: Mature Arabidopsis root at the stage of chemical and electron microscopic analyses, Table S1: ANOVA statistical test of the drought stress experiment, Table S2: ANOVA results of the salt stress experiment, Table S3: Primers used for PCR genotyping, Table S4: Data from ICP-MS analysis of elements in leaf tissues, Table S5: Results of the Kruskal-Wallis test investigating genotypic effect for leaf micronutrient content.
