# Supplementary material for: Root Suberin Plays Important Roles in Reducing Water Loss and Sodium Uptake in Arabidopsis thaliana
Source: Metabolites. 2021 Oct 27;11(11):735. doi: 10.3390/metabo11110735 (PMC8618449; doi:10.3390/metabo11110735)
Supplement: Supplementary file 1 [file metabolites-11-00735-s001.zip › metabolites-1352517-supplementary.pdf]

metabolites

Root suberin plays important roles in reducing water loss and sodium uptake in *Arabidopsis thaliana*

Nayana D G de Silva [1], Jhadeswar Murmu [1], Denise Chabot [2], Keith Hubbard [2], Peter Ryser [3], Isabel Molina [4,*] and Owen Rowland [1,*]

Supplementary

Table S1. ANOVA statistical test of the drought stress experiment. Total dry mass (TDM), root mass ratio (RMR), relative water content (RWC), and root suberin were considered as the dependent variables, and genotypes (WT and mutants) and the two levels of water treatment (control and drought stress) as the independent variables. ***p < 0.001; **p < 0.01; *p < 0.05.

			F-ratio		
Trait	R^2	*n*	Genotype	Water level	Genotype x Water level
TDM	0.81	97	41.1^{***}	202.8^{***}	4.3^{**}
RMR	0.40	97	3.3^{*}	41.4^{***}	0.9
Leaf RWC	0.39	93	1.9	36.1^{***}	2.7^{*}
Root suberin	0.92	45	61.8^{***}	86.2^{***}	3.9^{**}

Table S2. ANOVA results of the salt stress experiment. Total dry mass (TDM), root suberin content, leaf Na and K content, and K/Na ratio as the dependent variables, and genotypes (WT and mutants) and the two levels of salt treatment (control and 100 mM NaCl) as independent variables. Data for *abcg2-1 abcg6-1 abcg20-1* are not included in the statistical analysis. ***p < 0.001; **p < 0.01; *p < 0.05

			F-ratio		
Trait	R^2	*n*	Genotype	100 mM NaCl treatment	Genotype x Treatment
TDM	0.61	79	2.7^{*}	101.4^{***}	0.7
Root suberin	0.91	36	86.9^{***}	0.2	5.3^{**}
Leaf Na	0.90	40	8.2^{***}	261.3^{***}	2.6^{*}
Leaf K	0.75	40	16.8^{***}	36.9^{***}	4.1^{**}
K/Na ratio	0.93	40	11.9^{***}	443.3^{***}	0.2

Table S3. Primers used for PCR genotyping.

**Mutant**	**Publication**	**Locus and T-DNA or transposon insertion line**	**Primer Name and sequence (5'-3')**
cyp86b1-1	Compagnon *et al.,* 2009	AT5G41040; SM_37066	LS 331 (LP): TCCATCAGGAAATACGTCGTC<br>LS 332 (RP): CCTACTTGCGTGTGGAAGTTC<br>LS 186 (LB): TACGAATAAGAGCGTCCATTTTAGAGTGA
cyp86b1-2	Compagnon *et al.,* 2009	AT5G41040; SALK_130265	HS 101 (LP): GGTTTAGCAGCCTCAACAGC<br>HS 102 (RP): ATCTGGACCGAGACATCCTG<br>HS 100 (LB): TGGTTCACGTAGTGGGCCATCG
far1-2	Domergue *et al.,* 2010	AT5G22500; SALK_149469	FAR1-2 (LP): TGTTGCAATAAATGAAATGAACAG<br>FAR1-2 (RP): TACCTTGCACGACTATGTCCC<br>LBb1: GCGTGGACCGCTTGCTGCAACT
far4-1	Domergue *et al.,* 2010	AT3G44540; SALK_000229	FAR4-1 (LP): TGTATTCATCAAACCAATTGATCC<br>FAR4-1 (RP): TTGCGATGGTGAACTACTTCC<br>LBb1: GCGTGGACCGCTTGCTGCAACT
far5-1	Domergue *et al.,* 2010	AT3G44550; SALK_152963	FAR5-1 (LP): TTCTTGCAACGTCCTTAGCTG<br>FAR5-1 (RP): AAAGGTGGTATATAAAATTTCTTGTAGC<br>LBb1: GCGTGGACCGCTTGCTGCAACT
abcg2-1	Yadav *et al.,* 2014	AT2G37360; GABI_036B02	LP-GABI_036_B02: GAAGTTTAATCCCCTCGCTTG<br>RP-GABI_036_B02: CCTTTTGGGGAATTGTCTAGG<br>LBb1: GCGTGGACCGCTTGCTGCAACT
abcg6-1	Yadav *et al.,* 2014	At5G13580; SALK_ 050113	LP-Salk_050113: GATGCTGGTGGTGACTACGAC<br>RP-Salk_050113: TCAGGACATAAAACCTGGTGG<br>LBb1: GCGTGGACCGCTTGCTGCAACT
abcg20-1	Yadav *et al.,* 2014	At3G53510; SALK_011548C	LP-Salk_011548C: GTTGGAAATCCAATTAACCCC<br>RP-Salk_011548C: TTGAAATCCGATTGGCTAATG<br>LBb1: GCGTGGACCGCTTGCTGCAACT
myb92-1	This study	AT5G10280 SM_3_41690	LP-SM_3_41690: GGGTAGGTTTTCTCTTTGAGTGG<br>RP-SM_3_41690: CAGTTAGTGGTTGTGAAGGAAGG<br>Spm32_R (Lb): TACGAATAAGAGCGTCCATTTTAGAGTGA
myb93-1	This study	AT1G34670 SALK_131752	LP-Salk_131752: TTTAAGAGGTTCATGGCATGG<br>RP-Salk_131752: GGCTTCGTCGCTAGCTAGAAG<br>LBb1: GCGTGGACCGCTTGCTGCAACT

Table S4**.** Data from ICP-MS analysis of elements in leaf tissues. *Abcg2-1 abcg6-1 abcg20-1* data are not included for NaCl treatment due to salt hypersensitivity. For each element, mean values are from 4 replicate samples ± SE. Each replicate represents a pool of 4-5 leaves from an individual plant. Concentrations are given in parts per million (ppm) ± SE under control (0 mM NaCl) and 100 mM NaCl treatments. Data in bold represents significant differences between control and NaCl treatment in each genotype at p<0.05 by Student's t-test. Asterisks indicate differences between wild-type control and mutant control or NaCl treated plants at p<0.05 by Student's t-test.

	Wild-type (Col-0)		*abcg2-1 abcg6-1 abcg20-1*	*far1-2 far4-1 far5-1*		*cyp86a1-1 cyp86b1-1*		*myb92-1 myb93-1*	
Element	Control	NaCl (100mM)	Control	Control	NaCl (100mM)	Control	NaCl (100mM)	Control	NaCl (100mM)
Na	2383.15±421.3	**24319.16±6646.9**	13626.72±2909.3	2208.46±723.1	**24240.87±7979.2**	3351.56±560.6	***64392.33±25442.0**	1834.52±288.7	**39941.14±25507.2**
K	34334.085±1908.6	**26253.93±3935.0**	13423.18±1892.6	27132.87±3592.4	**23250.75±3462.4**	26565.26±4095.1	***14219.72±3953.1**	22492.32±2777.2	19991.80±3616.2
Mg	1471±158.0	2412±263.6	1859±186.4	1397±218.7	26439±273.8	1420±86.8	2110±226.7	1248±207.3	2567±241.9
Ca	19166±716.8	19331±1202	**21200±956**	16587±688	**21061±1462**	16930±1802	**20648±697**	*13477±751	**20122±36**
Cd	0.08±0.0	**0.45±0.0**	0.05±0.0	0.07±.0.00	**0.47±0.0**	0.06±0.0	**0.56±0.0**	0.05±0.0	**0.37±0.0**
Mo	2.51±0.3	**1.52±0.1**	2.44±0.2	2.73±0.4	1.92±0.4	2.21±0.3	1.75±0.1	2.20±0.2	1.67±0.7
Zn	25.8±0.6	**41.6±2.3**	28.8±2.1	23.9±0.7	39.6±3.3	24.0±0.4	**35.2±0.7**	*20.3±1.2	**36.3±2.1**
Si	91.8±7.8	**65.2±9.1**	111.8±15.2	130.3±27.8	108.8±15.2	79.3±5.6	76.8±10.9	61.6±16.4	47.2±3.3
B	44.2±2.5	47.5±2.9	57.7±13.9	*29.8±1.1	**25.9±1.4**	*28.5±2.3	**19.2±0.2**	*27.7±0.4	26.8±2.4
Li	0.17±0.0	**0.04±0.0**	0.08±0.0	*0.50±0.1	**0.04±0.0**	0.42±0.2	0.15±0.0	0.26±0.1	**0.08±0.28**
Al	153.94±30.5	**53.50±13.7**	*52.74±8.3	*507.69±165.4	**40.16±8.5**	*410.07±120.7	**108.61±20.9**	*269.25±84.8	76.0±7.9
Cr	1.48±0.3	1.10±0.1	1.15±0.1	1.11±0.0	1.21±0.1	1.38±0.1	1.31±0.1	1.08±-.1	1.14±0.4
Mn	175.95±11.6	**118.13±10.7**	146.84±6.1	145.27±10.2	**116.90±10.6**	186.92±21.4	**124.43±10.6**	123.49±7.8	**97.96±2.3**
Fe	422.50±98.6	**207.39±40.3**	*230.31±14.8	*792.85±165.6	**183.81±12.6**	*1017.88±480.9	317.09±72.8	*689.12±197.9	242.87±12.2
Co	1.13±0.04	1.56±0.3	0.98±0.1	1.30±0.05	1.24±0.1	1.28±0.1	0.88±0.1	1.03±0.1	1.19±0.5
Ni	0.88±0.12	0.88±0.13	0.78±0.04	0.75±0.04	0.84±0.1	1.11±0.1	0.88±0.1	0.86±0.1	0.91±0.4
Cu	8.73±0.2	8.24±0.16	9.70±0.7	8.23±0.3	8.85±0.3	8.36±0.3	8.39±0.5	6.54±0.5	**7.61±0.8**
As	0.07±0.01	0.06±0.0	0.05±0.0	0.06±0.0	0.06±0.0	0.07±0.0	0.07±0.0	0.04±0.0	0.06±0.1
Se	0.27±0.0	0.09±0.0	0.21±0.1	0.13±0.1	0.17±0.0	0.26±0.1	0.08±0.0	0.11±0.0	0.11±0.0

Table S5: Results of the Kruskal-Wallis test investigating genotypic effect for leaf micronutrient content. The significance at $p<0.05$ (*) is indicated by asterisks.

Element	p-value
Li	0.340
B	0.000*
Al	0.408
Si	0.001*
Cr	0.210
Mn	0.030*
Fe	0.580
Co	0.130
Ni	0.190
Cu	0.010*
Zn	0.340
As	0.070
Se	0.380
Mo	0.690
Cd	0.260
Ca	0.560
Mg	0.870

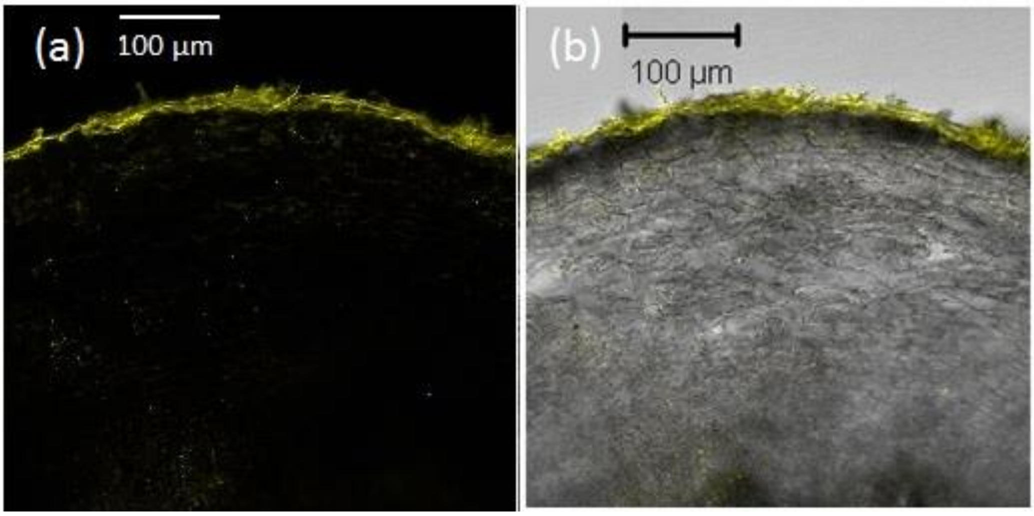

Figure S1. Microscopic view of suberin distribution in wild-type Arabidopsis roots under drought conditions. (a) Confocal laser scanning microscopy of a root periderm cross-section represented after staining with 0.01% Fluorol Yellow 088 (Brundrett *et al.*, 1998) [60]. Confocal settings were: 365 nm excitation filter, Ft 395 chromatic beam splitter and LP 420 nm emission filter. (b) Overlay of (a) and Differential Interference Contrast (DIC) microscopy for better observation of the surrounding tissues.

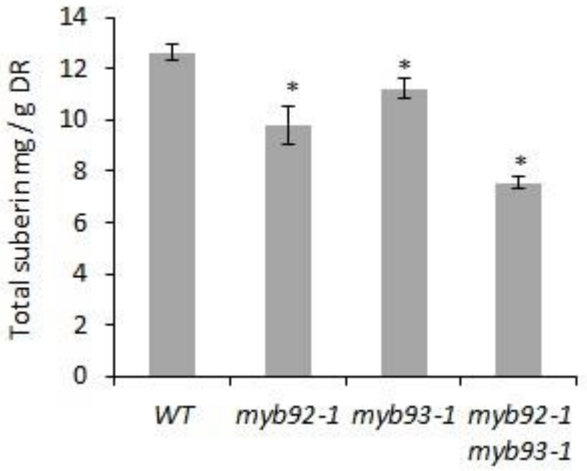

Figure S2. Total poly-aliphatic suberin in roots of wild-type, *myb92-1*, *myb93-1* and *myb92-1 myb93-1*. Mean values of total suberin are given in milligrams of suberin per gram of delipidated dry residue (DR) from 3-4 replicate samples ± SE. Each replicate consisted of roots from four pooled plants at 4-weeks old. Asterisks indicate significant differences by Student's t-test at $p<0.05$ comparing wild-type (WT) and mutant.

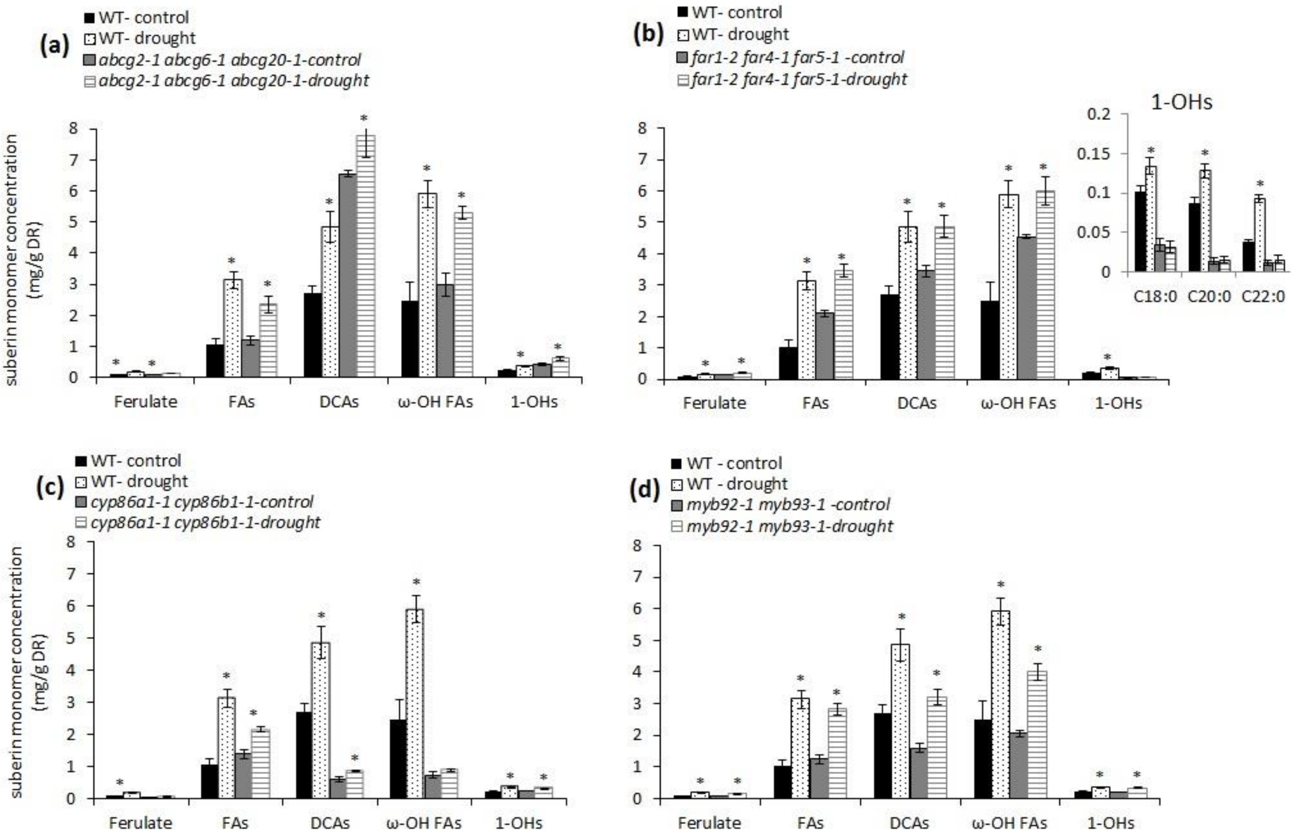

Figure S3. Root suberin composition under control and drought stress in wild-type and suberin mutants. (a) *abcg2-1 abcg6-1 abcg20-1*, (b) *far1-2 far4-1 far5-1* (inset shows changes in fatty alcohol composition), (c) *cyp86a1-1 cyp86b1-1*, and (d) *myb92-1 myb93-1*. Mean values are shown in milligrams of suberin monomers per gram of delipidated dry residue (DR) from 3-4 replicate samples ± SE. Each replicate represents roots from 4 pooled plants that were 4-weeks old. FAs, fatty acids; DCAs, dicarboxylic fatty acids; ω-OH FAs, hydroxy fatty acids; 1-OHs, primary alcohols. Asterisk indicates $p<0.05$ by Student's t-test comparing control and drought stressed plants.

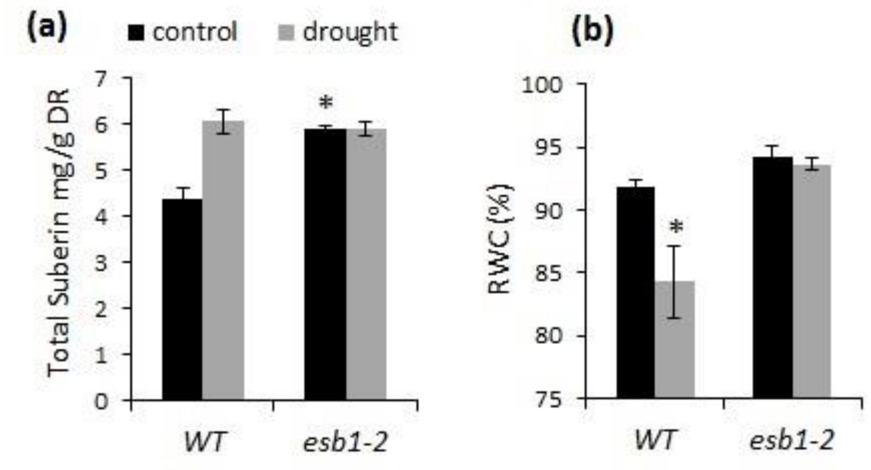

Figure S4. Comparison of root suberin and leaf relative water content (RWC) in wild-type and *esb1-2* under control and drought conditions. (a) Total suberin content. (n=4-5, with each replicate representing roots from 4 pooled plants at 4 weeks of age). Asterisk indicates the significance at $p<0.05$ in the difference in mean by Student's t-test comparing the control plants of wild-type and *esb1-2*. Values represent mean ± SD. (b) leaf relative water content (RWC) of wild-type and *esb1-2* under control and drought stress (n=10, with each replicate representing one leaf of an individual plant). Asterisk indicates the significance at $p<0.05$ in the difference in means by Student's t-test comparing control and drought-stressed plants.

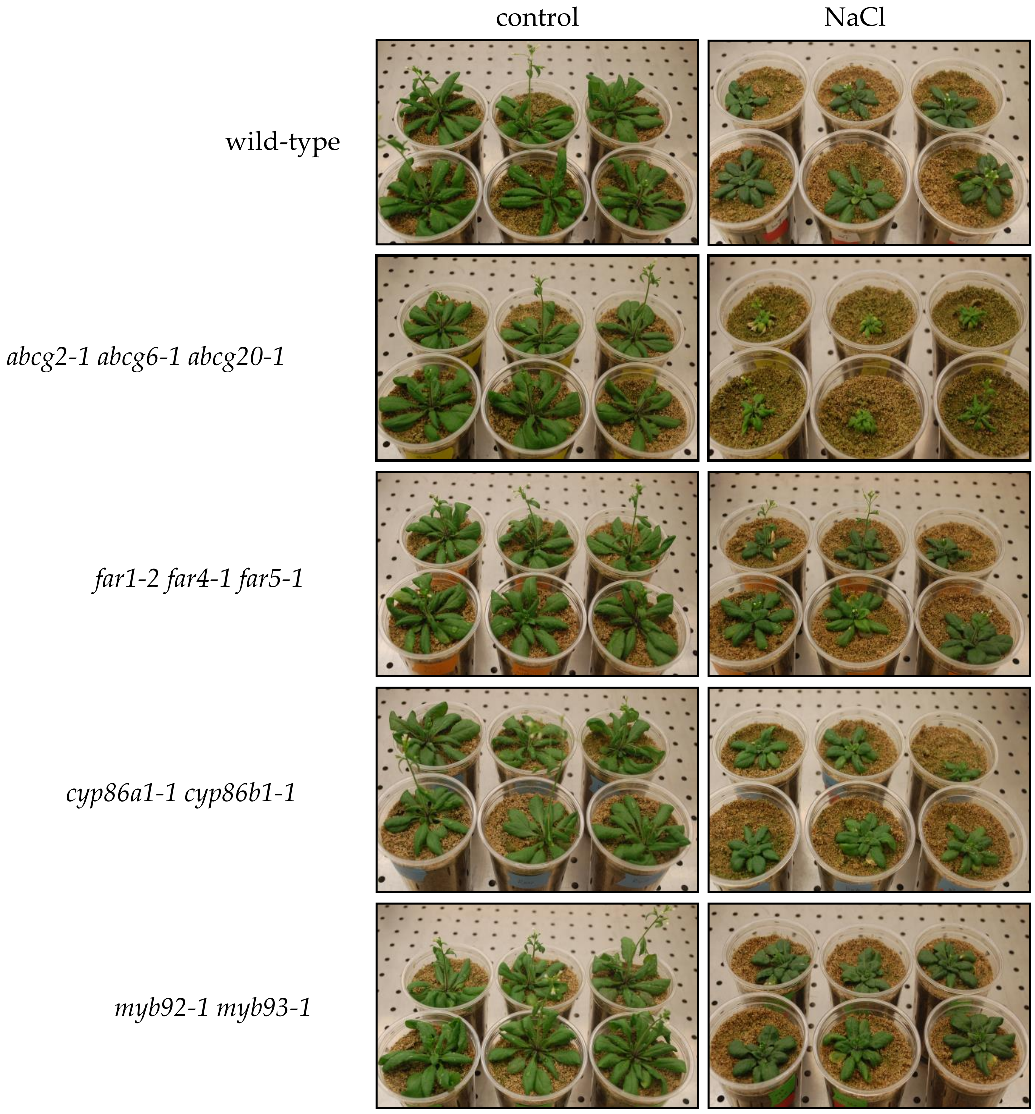

Figure S5. Comparison of plant sizes of control (0 mM NaCl) and after treatment with 50 mM or 100 mM NaCl for 2 weeks. Wild-type, *far1-2 far4-1 far5-1*, *cyp86a1-1 cyp86b1-1* and *myb92-1 myb93-1* plants were exposed to 100 mM NaCl and *abcg2-1 abcg6-1 abcg20-1* was exposed to 50 mM NaCl. Reduced plant sizes were observed in all mutants under NaCl treatment compared to control conditions. The *abcg2-1 abcg6-1 abcg20-1* mutant treated with 50 mM NaCl was smaller relative to the other mutants exposed to 100 mM NaCl.

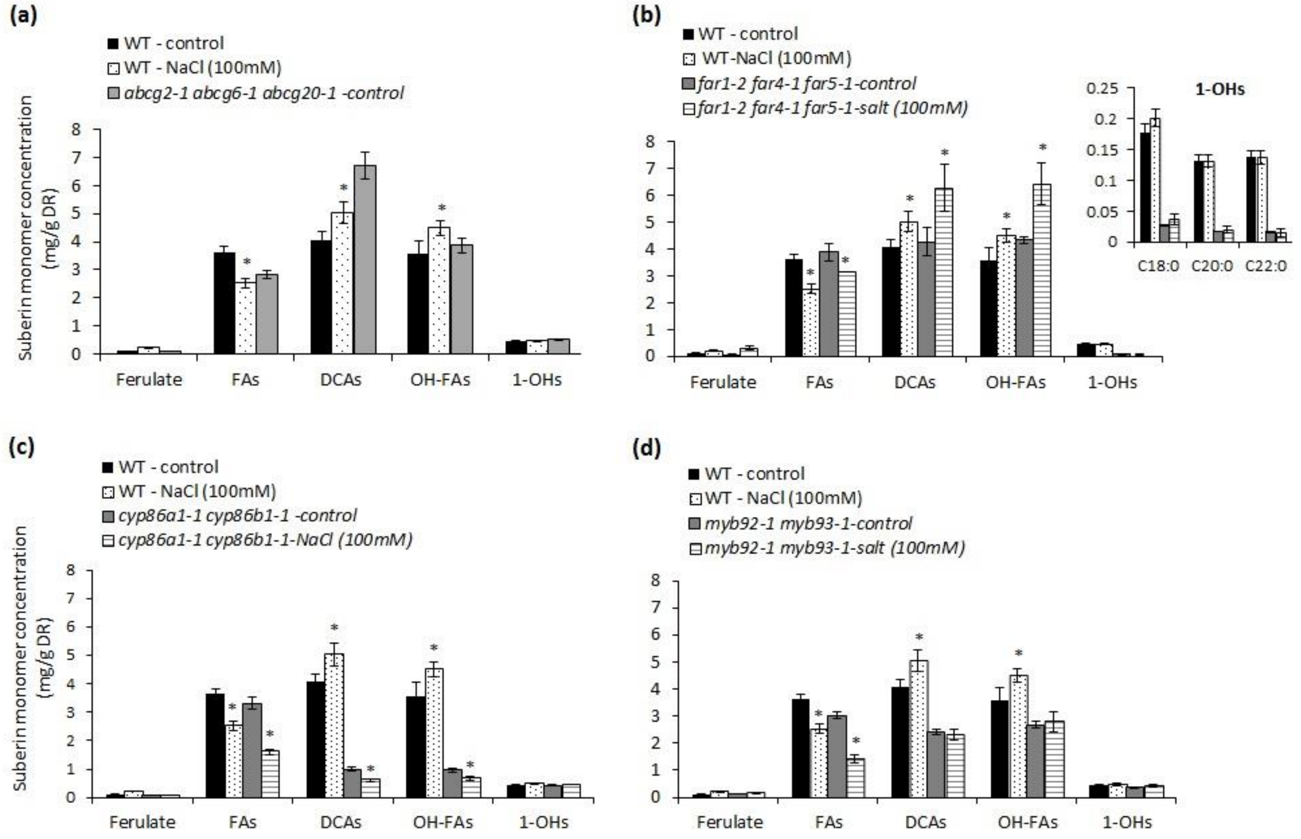

Figure S6. Comparison of root poly-aliphatic suberin composition in mutants and wild-type under control and NaCl treatments. (a) *abcg2-1 abcg6-1 abcg20-1*, (b) *far1-2 far4-1 far5-1* (inset showing changes in primary fatty alcohol composition), (c) *cyp86a1-1 cyp86b1-1* and (d) *myb92-1 myb93-1*. *Abcg2-1 abcg6-1 abcg20-1* data for 100 mM NaCl treatment are not included as this mutant incurred a high rate of mortality. Mean values are shown in milligrams of suberin monomer per gram of delipidated dry mass from 3-4 replicate samples ± SE. Each replicate represented roots from 4-5 plants that were 4-weeks old. FAs, fatty acids; DCAs, dicarboxylic fatty acids; ω-OH FAs, omega hydroxy fatty acids; 1-OHs, primary alcohols. Asterisk indicates $p<0.05$ by Student's t-test comparing control and drought stressed plants.

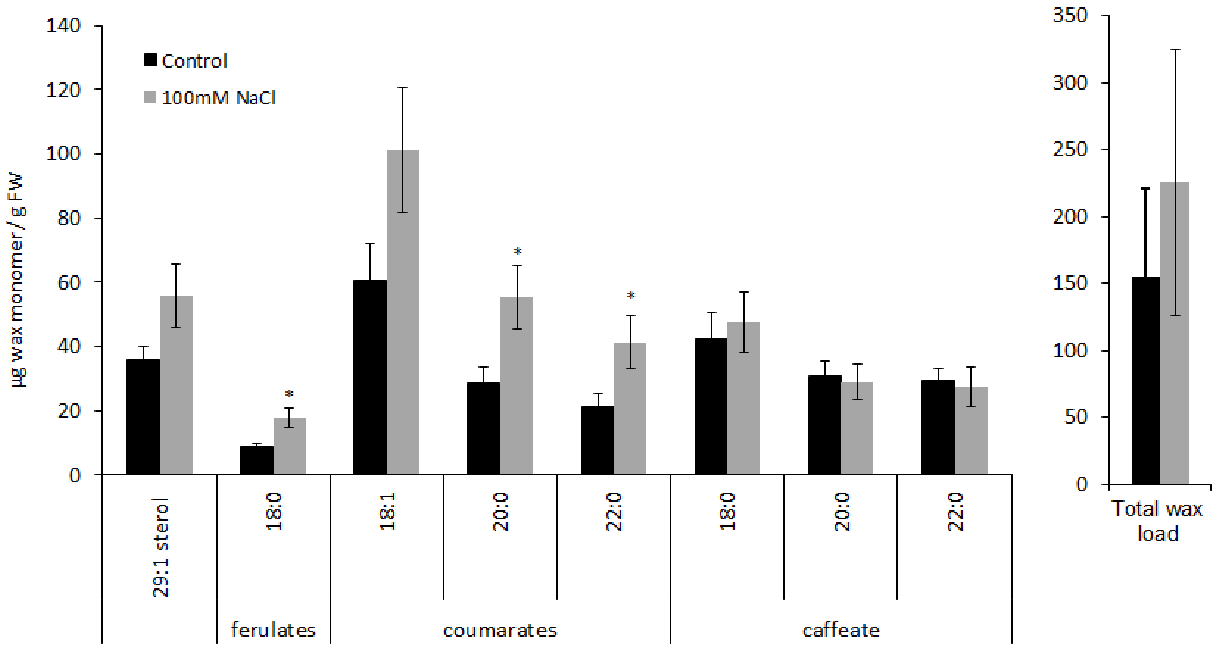

Figure S7. Comparison of root suberin-associated wax composition in wild-type plants under control conditions and after 3 weeks of 100 mM NaCl treatment. Mean values are shown in micrograms suberin monomer per gram of fresh weight from 3-4 replicate samples ± SE. Each replicate represented 10 root periderm segments of separate plants. Asterisks indicate $p<0.05$ by Student's t-test comparing the values of the control plants.

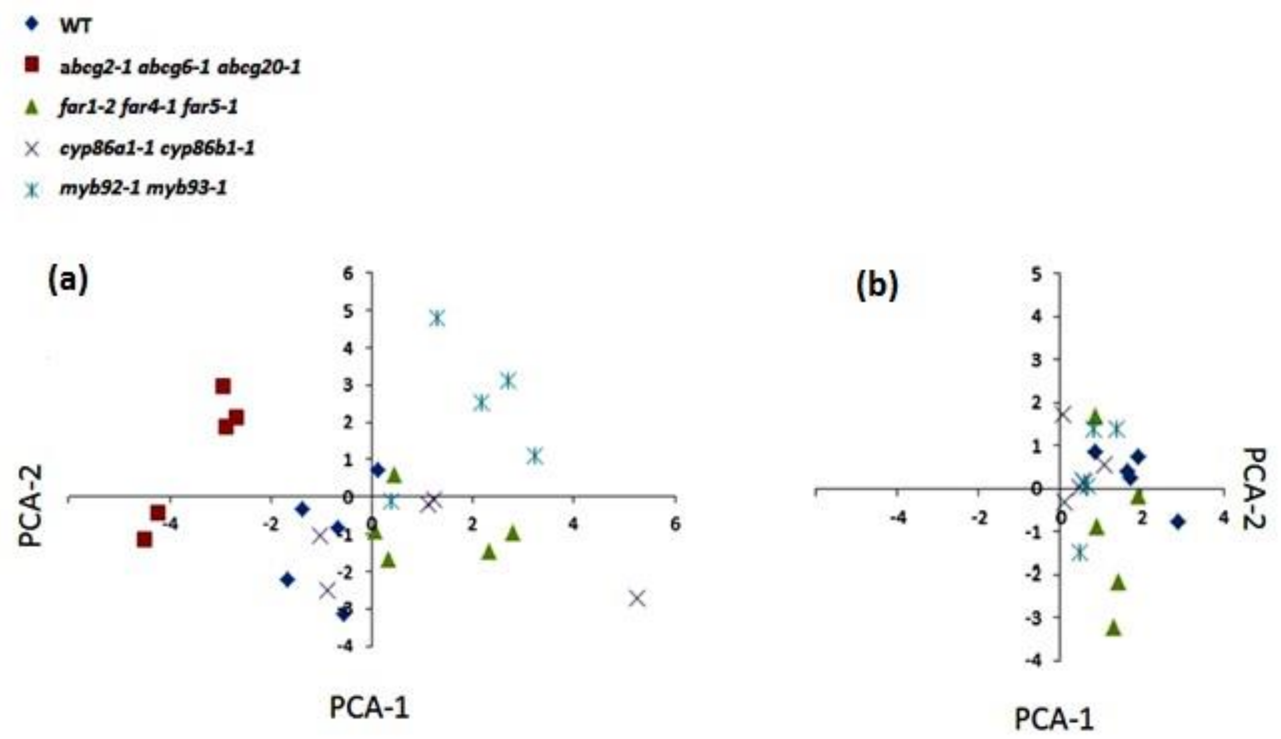

Figure S8. Segregation of leaf ionomic phenotypes in wild-type and mutants by Principal Component Analysis (PCA). (a) plants under control condition, (b) plants after 100 mM NaCl treatment. The PCA is based on the leaf concentrations of all the elements listed in Table 3 for wild-type and mutants. The analysis was performed on data from n=10 plants for each genotype and each treatment. *Abcg2-1 abcg6-1 abcg20- 1* mutant was excluded from the PCA analysis for NaCl treatment as it incurred a high rate of mortality. a) 30% variance in x-axis (PCA1) and 21% variance in y-axis (PCA2); in b), 43% variance in x-axis (PCA1) and 14% variance in y-axis (PCA2). There is a separation of data in *abcg2-1 abcg6-1 abcg20-1* from other genotypes under control conditions.

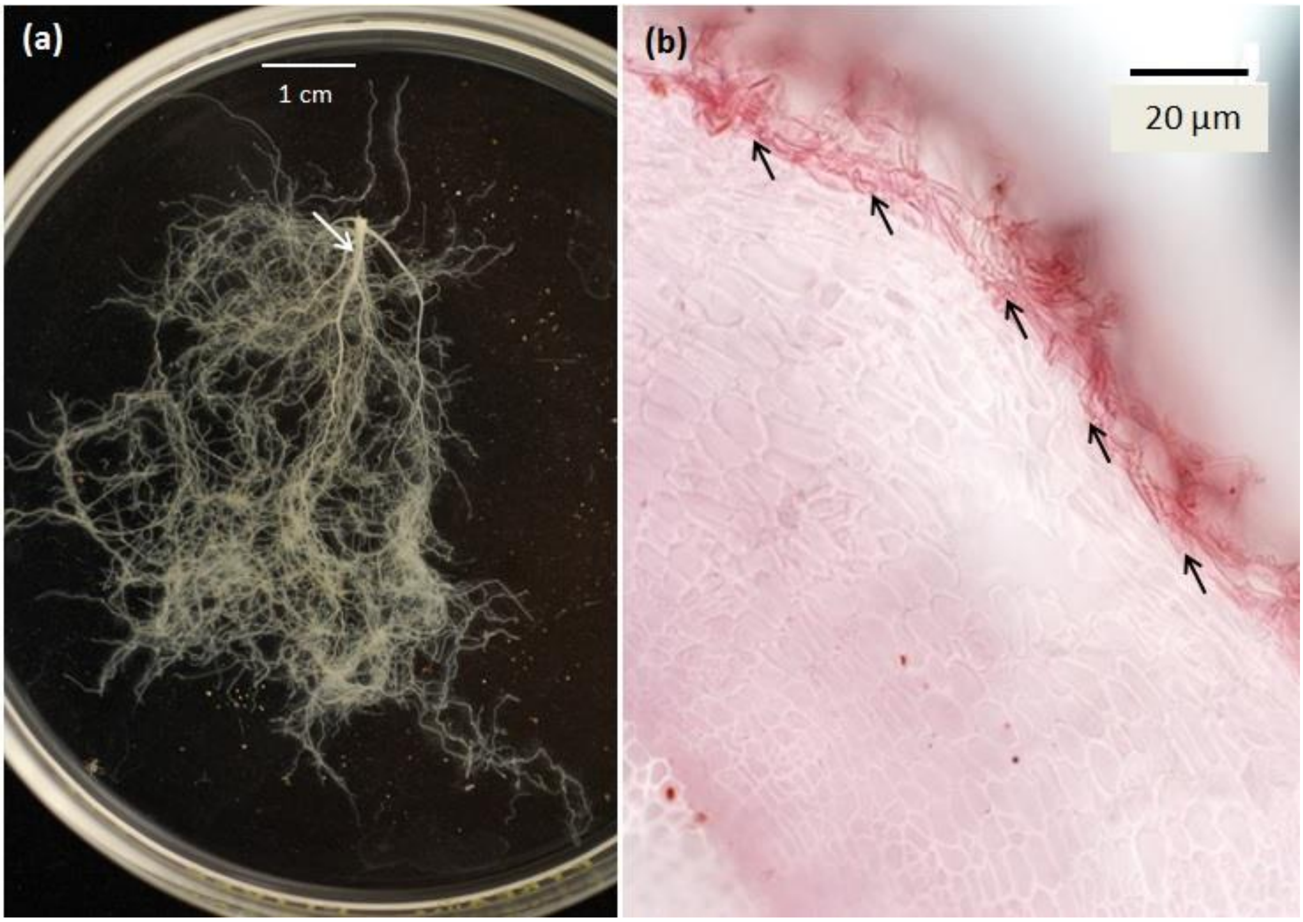

Figure S9. Mature Arabidopsis root at the stage of chemical and electron microscopic analyses. (a) Intact root of 4-week-old wild-type plant that had been undergone secondary development showing mature tap root (white arrow) at the harvest / chemical analysis / imaging stage. (b) Bright field light microscopic view of tap root cross-section showing suberin containing periderm tissue (black arrows). Cross sections were taken 1 mm below the hypocotyl junction and stained with Sudan red.
